# Dosimetric Implications of Number of Breathing Phases Used in the Definition of Internal Target Volume [ITV] in the Treatment of Non-Small Cell Lung Cancers Using Stereotactic Body Radiation Therapy (SBRT)

**Published:** 2019-10-05

**Authors:** Von Darius Heard, Eftekhar Rajab Bolookat, Bradley Rauschenbach, Kate Martin, Jorge Gomez, Anurag K Singh, Harish Malhotra

**Affiliations:** Department of Radiation Medicine, Roswell Park Cancer Institute, USA

**Keywords:** Stereotactic body radiation therapy, Non-small cell lung cancer, Respiratory gating, Internal target volume

## Abstract

Determination of intrafraction motion in stereotactic body radiation therapy (SBRT) of non-small-cell lung cancer (NSCLC) usually involves generating an internal target volume (ITV) based on target segmentation in every one of the 10 phases of a 4-dimensional computed tomography (4DCT) dataset which increases dosimetry work load substantially. This study explores the feasibility of using a smaller number of phases to compile an ITV to get equivalent results.

Twenty-five lung cancer patients treated with SBRT were retrospectively assessed. Patients were categorized by the anatomic location of the GTV within different lobes of the lungs, 5 in each lobe. Ten GTVs were contoured by the radiation oncologist in 10 different phases of one complete respiratory cycle. Five samples (Sample 1–5) were created using (0%, 20%, 40%, 60%, 80% i.e. taking every other phase), (0%, 30%, 60%, 90% i.e. skipping two successive phases), (0%, 20%, 30%, 50% i.e. essentially taking inhale, exhale & a phase in between), (0%, 30%, 60%), (0%, 50% i.e. using completely inhale and exhale phase only) phase GTVs, 0% is designated as the most inhaled phase and 50% as the most exhaled phase. Appropriate sample ITVs and PTVs were created in the same manner as the clinical plan which was then adapted to each sample set with minimal modification. Sample plans were compared for equivalent dose coverage, center of mass, and ITV/PTV volume differences against the clinical treatment plan.

The average % ITV underestimation against the clinical ITV increased from a minimum of 7.3% in sample 1 (0%, 20%, 40%, 60%, 80%) to a maximum of 24.5% in sample 5 (0% & 50%) under the conditions of controlled breathing. A similar trend was seen in other samples with the underestimation in the ITV/PTV volume increasing with the decrease in the number of phases irrespective of the tumor location. The average variation in the center of mass of the ITV was minimal (0.43 ± 0.11 mm). Use of ITV/PTV combination derived from using less than all 10 phases resulted in the maximum clinical PTV under-dosage of 5.9% for sample 1 and 12.3% for sample 5, respectively. If fewer phases in the generation of ITV are used, a larger ITV-to-PTV margin might be necessary to get equivalent PTV coverage.

## Introduction

**Figure 1:**
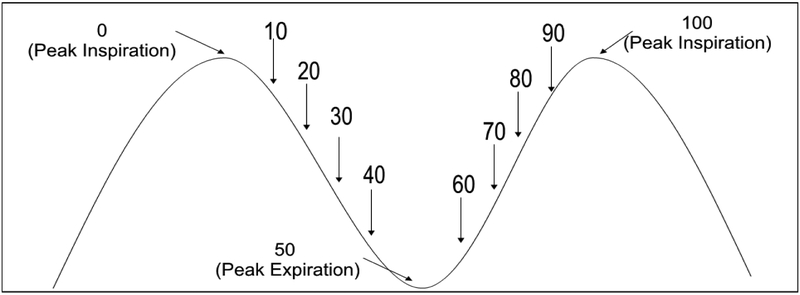
Schematic of a human breathing cycle in the form of a sinusoidal wave form. The numbers on the curve represent the location of various bins used to segregate the original 4DCT dataset with 0% & 50% representing maximum inhale and exhale states, respectively.

Stereotactic body radiation therapy (SBRT) is gaining wide clinical acceptance in the management of early non-small-cell lung cancer [1–3]. SBRT involves the delivery of ablative doses of external radiation therapy in a hypofractionated scheme, typically with 30–60 Gray (Gy) delivered in 1–5 fractions. Such high doses of radiation require precise and accurate delineation of the gross tumor volume (GTV) in order to protect surrounding critical structures while giving a tumorcidal dose to the entire tumor. This is usually best achieved through the acquisition of a four dimensional computed tomography (4DCT) scan to help in the delineation of an internal target volume (ITV) that will account for the intrafraction motion of the tumor [4]. The 4DCT dataset gathers spatial as well as temporal information of the tumor during the entire breathing cycle of the patient. Since computerized treatment planning systems can handle only a 3DCT dataset, the standard method to integrate temporal information is typically to break up the 4DCT data into a subset of 10 bins at 10% increments of the breathing cycle (Figure 1). The process of deriving an ITV which encompasses the entire intrafraction motion during a breathing cycle is carried out using a BOOLEAN OR operation on the tumor volumes segmented in each of the 10 phases. The entire process increases the dosimetry work load immensely [5]. The purpose of this study was to determine the feasibility of using a smaller number of phases to compile an ITV to get equivalent results as that obtained using 10 phases of breathing cycle to improve workflow efficiency and yet maintaining the required accuracy of planning design.

## Methods and Materials

### Patient data, Simulation and Clinical treatment planning

Twenty five patients (10 males, 15 females) who were previously treated for inoperable non-small cell lung cancer (NSCLC) using SBRT were retrospectively assessed in this study. The patients’ age ranged from 62–85 years with an average age of 75.8 years. Patients were categorized based on the lobe of the lung where the tumor was located (5 patients per lobe) viz. RUL, RML, RLL, LUL, LLL corresponding to right upper lobe, right middle lobe, right lower lobe, left upper lobe and left lower lobe, respectively.

Patients were simulated with their arms extended above their head lying on a customized thoracic blue bag immobilizer indexed to the CT table with an abdominal compression device applied below the xiphoid to decrease breathing motion. Two types of CT scans were taken using GE 16 slice scanner [GE lightspeed RT16] in conjunction with Varian’s real-time position management (RPM) respiratory gating system employing an external surrogate system to correlate with the tumor motion. The RPM system uses a light weight plastic cube with 2 infrared markers placed on the patient body, the motion of which is tracked in real time by an infrared camera system. A helical full scan from the level of the mandible to below the liver and included the tumor and critical structures in its entirety was acquired for the actual treatment planning. Slice thickness ranged from 1.25–2.5 mm. Then a shorter scan extending 5–7 cm superior and inferior the GTV was obtained in the 4DCT cine mode to capture the entire tumor excursion i.e. ITV. In 4DCT cine mode, CT images are acquired in conjunction with the RPM system with the table stationary for a specified time interval over the respiratory cycle of the patient after adding a small margin for the probable variation in the respiration. The table is then moved to the next position and the process is repeated till the entire volume is scanned. The entire process yields a large number of CT images [up to 3000], the exact number of which depends on the respiratory cycle of the patient, the slice thickness and slice width chosen, pitch of the scanner, region selected for scanning etc. These phase-based images are then retrospectively analyzed in concurrence with the RPM gating information, which binned the 4DCT scan images into 10 discrete phase bins of the breathing cycle (Figure 1) with 0% as the most inhaled phase and 50% being the most exhaled phase. These 10 CT data sets were sent to the Eclipse treatment planning system [Varian Medical System, Palo Alto, USA] where the GTVn [where n is the bin number of the breathing phase] were segmented in each phase using the same CT window and by the same physician. These CT data sets were co-registered with the earlier complete controlled breathing scan using the DICOM origin as the patient had not moved between the controlled breathing and 4DCT scan sets. Individual GTVn’s were then copied to the complete controlled breathing CT data set. An ITV was generated by applying BOOLEAN OR operation on all the individual GTVn’s representing each of the 10 bins. Then a planning target volume (PTV) was created by adding a MD specified margin to the ITV. The volume of patient’s ITV ranged from 3.2–14.9 cm^3^, median 5.6 cm^3^ with an average of 6.3 cm^3^. Respective values for PTV ranged from 16.0–49.7 cm^3^ with a median of 24.5 cm^3^ & an average of 26.8 cm^3^. A clinical treatment plan was then generated based on the constraints provided by the MD using numerous non-coplanar beams ranging from 9–15. The treatment fields were conformed to the PTV by fitting the multi-leaf collimator (MLC) to the PTV using the fit to structure tool along with 0–1 mm margin. A 3DCRT treatment plan was calculated using Eclipse TPS. In all the patients, 100% prescription dose covered at least 95% of the PTV volume and 90% prescription dose covered 99% of the PTV volume. The patients’ prescription dose ranged from 30–60 Gy delivered in 1 to 5 fractions. This treatment plan was evaluated and approved for treatment by the radiation oncologist and will be, hereafter, referred to as the clinical plan. Table 1 summarizes various parameters of the patients used in this study.

### Retrospective treatment planning

**Figure 2:**
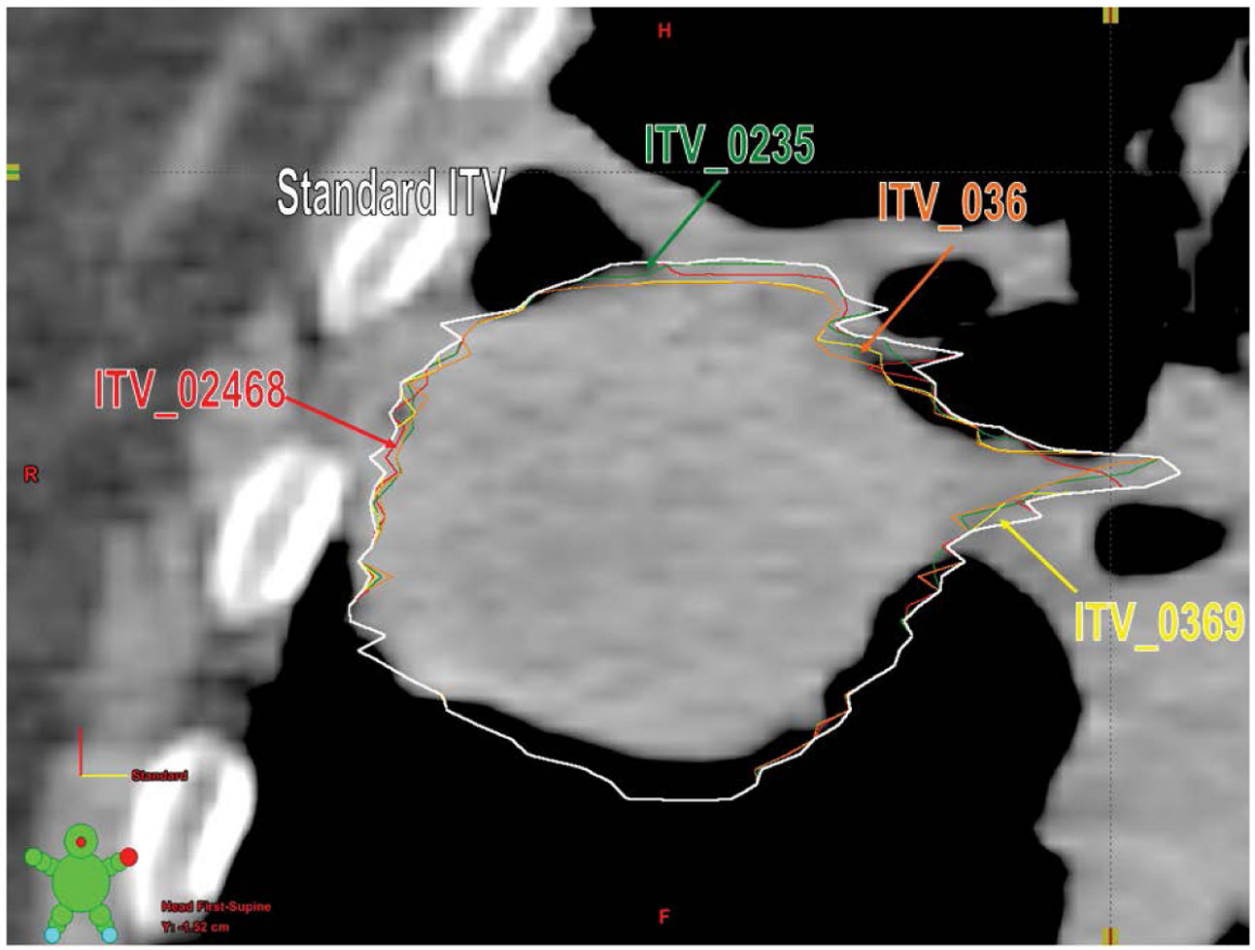
Transverse CT cut of a patient showing clinical ITV generated using all the 10 phases (Standard ITV). Also shown are the ITVs generated using a subset of all 10 phases represented as ITV_02468, ITV_0369, ITV_0235 and ITV_036 are the various samples which used (0%, 20%, 40%, 60, 80%), (0%, 30%, 60%, 90%), (0%, 20%, 30%, 50%) and (0%, 30%, 60%) to generate the respective ITVs.

Using the original 10 GTVs segmented by the physician, 5 sample ITVs were created. Sample 1 used GTVs from phases 0%, 20%, 40%, 60% and 80% (alternate phases) to create an ITV from every other phase of the breathing cycle. Sample 2 used GTVs from phases 0%, 30%, 60%, and 90% (basically skipping 2 successive phases after every phase) to create an ITV from every three phases of the breathing cycle (Figure 2). Sample 3 used GTVs from phases 0%, 20%, 30% and 50% to create an ITV using the peak inspiration phase (0%), middle of inhalation and exhalation phases (20% and 30%), and peak expiration phase (50%) of the breathing cycle. Sample 4 used GTVs from phases 0%, 30% and 60% to create an ITV from just three phases of the breathing cycle. Sample 5 used GTVs from only 0% and 50% phases essentially using the extreme inhale & exhale phases for ITV generation as is used in certain centers. Using the contouring workspace, the respective ITV’s were generated by BOOLEAN OR operation of the respective phases. Then 5 sample PTVs were generated from the 5 sample ITVs by using the same margin as in the original clinical treatment plan.

The original clinical plan was copied and pasted in each sample PTV defined earlier and these PTV volumes were then chosen as target for delivering the prescription dose. Various parameters like, center of mass (COM) and volume of the sample ITV and corresponding PTV were then obtained. Each sample was then calculated separately using the original clinical treatment plan with no changes in field arrangements, beam modifiers or heterogeneity corrections. The only difference was adjusting the MLC to fit the sample PTV as it was done in the clinical using the fit to structure tool in Eclipse treatment planning system. This resulted in a slightly different jaws and MLC positions when compared with the clinical plan. For a meaningful comparison, the prescribed percentage isodose line was adjusted to achieve the 100% prescription dose coverage to 95% of the PTV and 90% prescription dose coverage to 99% of the sample PTV as was in the clinical plan. This resulted in slight change in the prescription isodose line which ranged from −12.5% to 1.8% differences with an average of −1% difference. Sample plans were then compared with the clinical plan for volume [clinical PTV vs. sample PTV], COM, PTV prescription dose coverage and PTV 90% prescription dose coverage differences.

## Results

Table 2 gives the motion of the tumor along the 3-axis under the condition of controlled breathing using an abdominal compression device. Due to abdominal compression, a maximum motion of 9.2, 5 & 3.8 mm was noticed in the superior-inferior, anterior-posterior and lateral direction, respectively. Corresponding values of motion average (± SD) were found to be 3.8 ± 2.6, 2.4 ± 1.2 and 1.8 ± 0.8 mm along the superior-inferior, anterior-posterior and lateral direction, respectively. These low values demonstrate the advantage of a simple abdominal compression device to control tumor motion in studied cases. It is important to note that abdominal compression was tolerated well by the patients as this was customized for each patient.

**Figure 3:**
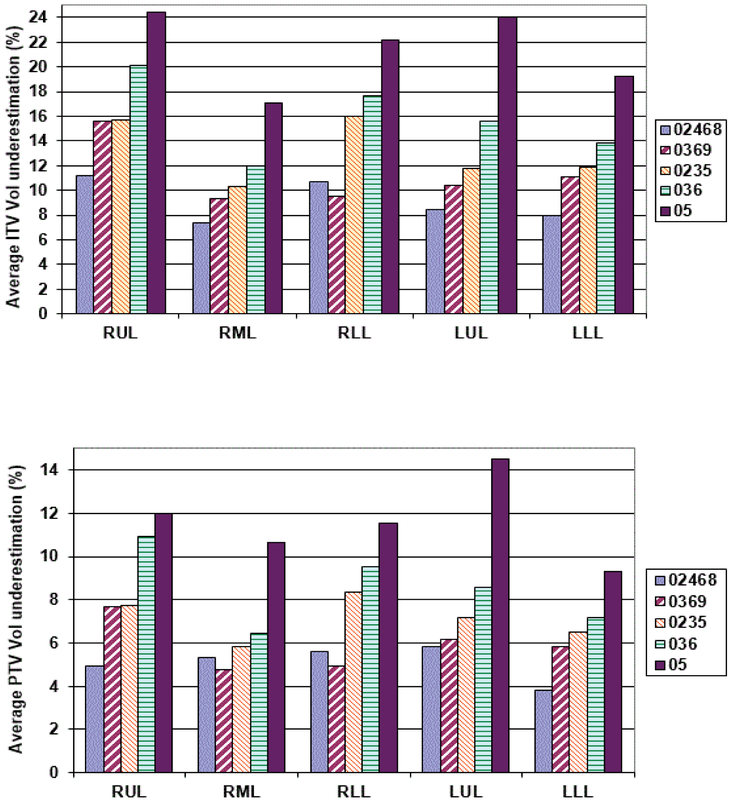
**a):** Average sample ITV underestimation against the ITV generated using all the 10 phases for various lung lobe tumors. 02468, 0369, 0235, 036 and 05 are the various samples which were used (0%, 20%, 40%, 60, 80%), (0%, 30%, 60%, 90%), (0%, 20%, 30%, 50%), (0%, 30%, 60%) and (0%, 50%) to generate the respective ITVs; **b)** Shows the same for the respective PTVs.

Figure 3 gives the average percentage ITV underestimation against the ITV obtained using all the 10 phases of 4DCT dataset. As it may be seen from (Figure 3a), all sample ITV’s generated using a subset of the complete 10 phases, would have led to an underestimation of ITV ranging from 1.7% to 34.7% depending upon the lung lobe where the tumor was located and the number of phases used in generating the ITV. Using every other phase (02468 sample) consistently produced maximum ITV underestimation (minimum 7.0%, maximum 10.7%) for all the lobes. A similar result was obtained for samples representing 0369 (0%, 30%, 60%) and 036 (0%, 30%, 60%) for all the lobes where the magnitude of ITV underestimation increased as the number of phases used to determine the ITV were reduced. Sample 0235 representative of full inhale (0%), mid level (20% & 30%) and full exhale (50%) CT sets used for ITV generation showed a minimum of 9.1% & a maximum of 16% underestimation. Sample 5 which just used phases in extreme inhale and exhale phases only showed a minimum of 18.2% with a maximum of 24.5% underestimation of the ITV. Similar underestimation in sampled PTV volumes was noticed when it was compared with the PTV generated using all 10 phases (Figure 3).

**Figure 4:**
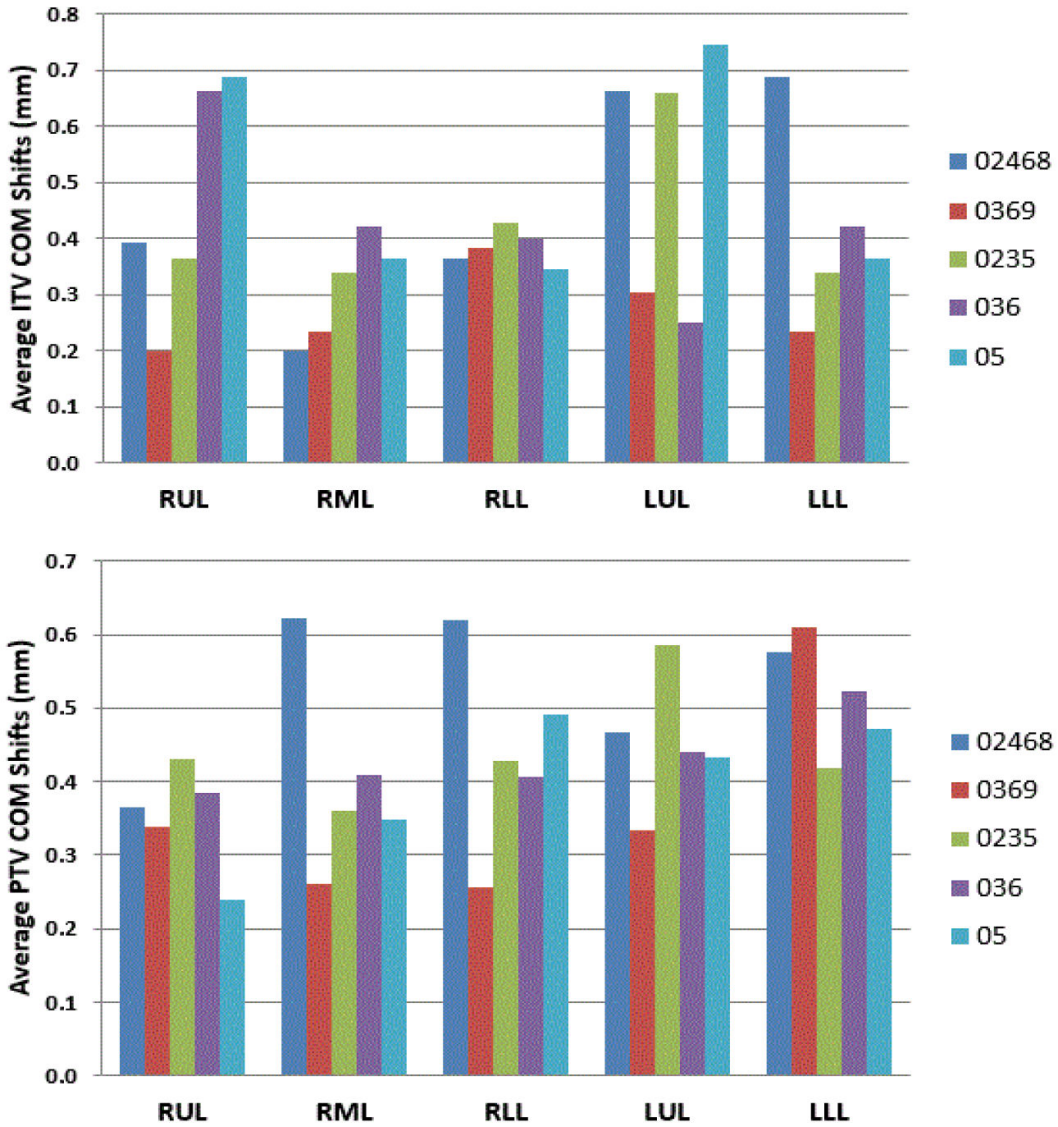
Variation of the center of mass (COM) of the sample PTV with respect to the clinical ITV & PTV (COM) in mm.

In SBRT planning, COM coordinates play an important role in defining the location of the isocenter using the PTV as the target structure. Thus a change in COM will lead to tumor targeting error if the COM values were generated using a PTV derived from a subset of phases. The variation in COM was studied in each of the three axis [x = lateral, y = anterior-posterior, z = superior-inferior direction]. The average variation in the sample COM coordinates with respect to COM coordinates determined using all the 10 phases was 0.43 ± 0.11 mm for all tumors irrespective of their location though a maximum shift of 1.9 mm was noticed in PTVs generated using a subset of phases (Figure 4).

**Figure 5:**
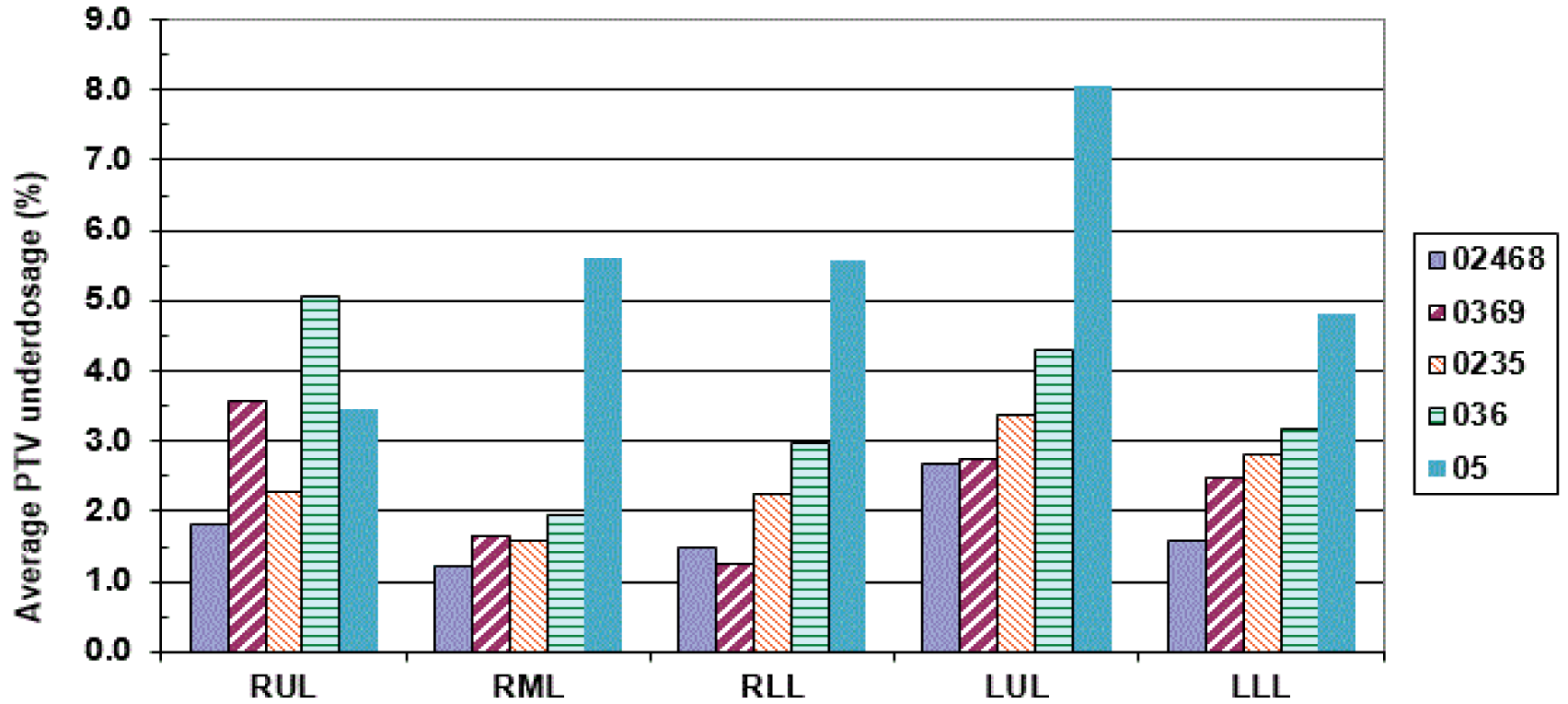
Graph showing the average reduction in the clinical PTV (using all the 10 phases) coverage of 95% volume generated getting the 100% prescription dose against the sample cases where subset of phases were used in ITV generation.

**Figure 6:**
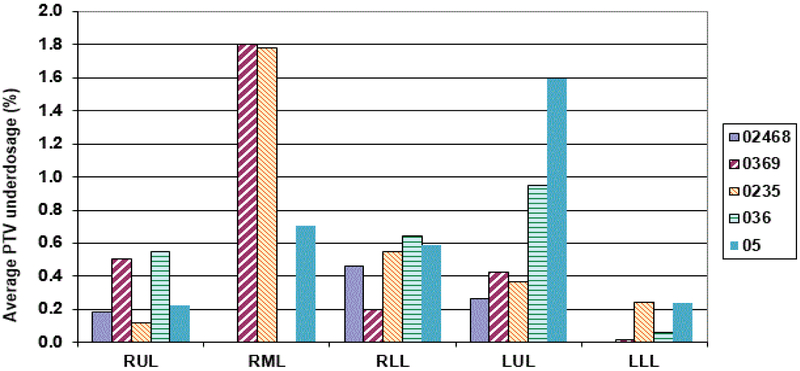
Graph showing the average reduction in the clinical PTV (using all the 10 phases) coverage of 99% volume generated getting the 90% prescription dose against the sample cases where subset of phases were used in ITV generation.

It is important to see how the prescribed dose coverage of the PTV is affected when the PTV is obtained using a subset of phases to generate the treatment plan rather than the complete set of ten phases. As already indicated, the samples had MLC leaves adjusted to the sample PTV with the identical margin (0–1 mm maximum) as was used in the original clinical plan. Furthermore, all the plans were normalized to deliver the prescription dose to 95% of the PTV volume to have an apple to apple comparison. The PTV coverage of the clinical plan was found to decrease up to 12.3% [i.e. only 87.7% of the clinical PTV is now getting prescription dose instead of clinical plan having 95% coverage] when the number of phases used for ITV excursion were reduced. When every other phase data (sample 0%, 20%, 40%, 60%, 80%) was used for ITV/PTV generation, a minimal average reduction in the clinical PTV receiving the prescription dose was noticed [minimum 1.2%, maximum 2.7%] (Figure 4). The maximum average reduction in the clinical PTV receiving the prescription dose was found for sample 5 (0%, 50%) which ranged from 5.9–12.3%. Another condition to evaluate adequate coverage of the PTV with SBRT states requires that 99% of the clinical PTV must receive a minimum of 90% of the prescribed dose as stipulated in various RTOG SBRT based clinical protocols like RTOG-0236. All the treatment plans generated using a subset of phases were also tested for this condition. We found that the maximum reduction in the clinical PTV coverage receiving 90% of the prescription dose was 9% (i.e. instead of 99% of clinical PTV, 91% of the clinical PTV still received 90% of the prescribed dose) (Figure 5 and Figure 6).

## Discussion

Lung tumors, unless they are attached to fixed bony structures like ribs etc, may demonstrate significant intrafraction motion during the entire breathing cycle, the magnitude of which also depends on the proximity of the tumor in relation to the diaphragm. This motion could be categorized as either voluntary or involuntary in nature. For a cooperative patient, voluntary motion could be suppressed by employing respiratory gating methods during simulation and dose delivery. Involuntary motion e.g. due to heart beats for tumors closer to heart, however, is difficult to control. Both voluntary as well as involuntary motions of the tumor could be suppressed by using a simple abdominal compression device, one of several techniques used to achieve controlled-breathing. Our experience has demonstrated that abdominal compression provides excellent reduction in the intrafraction motion, is well tolerated by the patients, allows reproducible compression between fractions and reduces tumor excursion within acceptable limits to treat most NSCLC cases without using respiratory gating techniques which though desirable, increases the overall treatment time and patient discomfort. This is often also compounded by the need to use a large number of non-coplanar fields which requires frequent re-tracking of the RPM gating system using the associated infrared markers.

Retrospective gating technique employing multi-slice CT scanners provide an efficient method of determining the tumor excursion in a patient. To make the process efficient, usually the 4DCT scans are binned in 10 uniform & discrete phases though it is possible to bin them in different number of bins as well. It is usually believed that there is only a minimal motion of a tumor between successive phases more so considering the fact that we have used abdominal compression in all the patients (Table 1). Omission of certain phases for generation of ITV, therefore, should be possible and should lead to minimal differences when compared to ITV’s generated from all the ten phases. However, our results show that this assumption may not be factual even when appropriate abdominal compression is in place regardless of tumor location within different lobes. Moreover, underestimation of ITV will increase if the number of phases used in the ITV generation is reduced. It will not be out of point to mention that all our results were with abdominal compression employed which significantly reduced the tumor motion. In those cases where such a device is not used, the true magnitude of difference when a subset of phases is used in ITV determination may even be higher. Since PTV depends on ITV, the error is propagated down the line.

One of the preferred approaches to treat these tumors is to determine their center of mass and use it to define the treatment plan isocenter. Since this center of mass is a function of PTV, a change of PTV by choosing less number of phases should impact its spatial coordinates as well. Thus, center of mass is an important parameter which will affect the targeting accuracy of the tumor. While significant differences between the PTVs were seen when the number of phases used for ITV definition were reduced, minimal differences in the spatial coordinates of the COM was noticed. This important finding implies that if due to operational reasons, a smaller number of phases were to be used to determine the ITV, it could be compensated by using slightly higher ITV to PTV margin.

With a view to reduce dosimetry segmentation times and yet to take advantage of 4DCT, the use of maximum intensity projection (MIP) scan dataset is described in literature [5–7]. A MIP CT dataset essentially compresses the temporal information from the 4DCT set and creates a single 3DCT data set based on the maximum intensity projection at a given axial scan level. In a study consisting of 12 stage I NSCLC patients, Underberg, et al. compared ITV generated using 10 bins of 4DCT with one generated using MIP and found good congruence between both implying that MIP provide a reliable and quick tool for generating ITVs [7,8]. It is important to know that if MIP dataset is used, there is no change either in the dosimetry work flow or time requirement for segmentations making this an extremely preferable approach in a clinical environment. However, this technique fundamentally relies on the fact that the tumor is a singular high density region surrounded by low density lung during the entire breathing cycle. This is difficult to achieve for tumors near the diaphragm or when the tumor is surrounded by other moving structures of equivalent density such as medium to large size blood vessels or where tumor is surrounded by atelectasis [5,7,8]. Since SBRT for NSCLC is no longer confined to peripheral lung lesions, the use of MIP to generate the ITV may not be a reliable method. In another study of 27 patients (stage I-III NSCLC), Ezhil, et al. compared ITV generated using all the 10 phases with ITV generated using MIP and they noticed that the ITV generated using MIP was significantly smaller than the one generated using all the 10 phases of breathing cycle regardless of the patient staging [9]. In another study, Muirhead, et al., studied 14 patients and compared the ITV generated using all the 10 phases vs MIP generated volumes and concluded that MIP ITV are smaller and hence may lead to underestimation [8]. They concluded that MIP generated ITV could be used only for the delineation of Stage I tumors. In a phantom study employing irregular target motion, Park, et al., noted that the MIP can underestimate target motion when the motion is irregular in amplitude and periodicity [10]. Similar results were noticed by another study by Huang, et al., where an under dosage exceeding 10% in the PTV was noticed for large and irregular tumor motions [11].

Attempts have also been made to generate ITV using the two phases [BOOLEAN OR] in which tumor is in its most superior and inferior position [12,13]. The major concern with this method is that tumors may not be moving linearly between two extreme phases and hence the ITV generated may be underestimated leading to geographical misses in certain phases of the breathing cycle. Moreover, lung tumors also under go deformation during their motion in breathing trace. In another study, Bosmans, et al., used 4DCT data set to determine the phase where the tumor is at its mid position and followed it up with the motion of tumor in all the three planes [14]. These margins are then used to enlarge the GTV derived from the tumor mid position scan to generate the necessary ITV. Since treatment planning systems can handle only maximum of two margin values along an axis, the user ends up using the maximum displacements and hence this method may over-estimate the resultant ITV because not all parts of the tumor may move by the same amount in one direction or there may be some deformation of the tumor during the breathing cycle as well. In another study, Wolthaus, et al. have proposed a method which generates a single 3D CT dataset from a 4DCT dataset representative of tumor in its time-averaged position over the respiratory cycle [15].

There are many others methods proposed in literature to determine the ITV for these type patients viz. incorporating different imaging techniques for individualizing margins using a slow CT [16], end-tidal breath-hold CTs [17], composite of two different helical scans in maximal inhale and exhale and breath-hold CTs [18]. No single method has yet been found to be universally acceptable as each one of them has its own limitations. Thus, it can be concluded that only when the appropriate factors are present, alternative methods of generating ITV using a subset of the original 4DCT dataset as proposed in the literature may provide equivalent results as that of ITV generated using all the 10 phases. ITV generated using all the 10 phases of a 4DCT dataset thus remains the gold standard for planning of NSCLC tumors using SBRT. Our results from this paper have supported this observation.

## Conclusion

Intrafraction motion in lung tumors is a cause of concern clinically. Due to the high dose of XRT per fraction when SBRT is used, reducing the PTV margins to cover the tumor adequately, thereby, reducing the irradiated volume is of the utmost importance. None of the ITV samples generated using a subset of phases, provided equivalent results as that used in a clinical plan utilizing all 10 phases for the determination of ITV. Even though using all 10 phases for the determination of ITV is more time-consuming & laborious, it might be the most beneficial method to ensure PTV coverage. Significant underestimation of ITV/PTV is possible even under controlled breathing setup employing abdominal compression if only a subset of phases are used for ITV generation.

## Figures and Tables

**Table 1:** Summary of the various clinical & dosimetric pertinent facts about each case used in the study. The tumor motion as measured after binning into 10 phases is also shown. The patient was positioned using an abdominal compression device.

Patient	Sex	Age (y)	Lung Lobe	ITV (cm ^3^)	PTV (cm^3^)	Lateral motion (mm)	Superior/Inferior motion (mm)	Anterior/Posterior motion (mm)
**1**	M	77	RUL	4.42	15.96	2.0	1.8	3.9
**2**	M	75	RUL	4.77	23.73	3.8	2.1	4.4
**3**	F	70	RUL	4.59	24.39	2.3	1.4	4.6
**4**	F	77	RUL	7.38	32.99	1.9	3.2	1.4
**5**	F	80	RUL	3.90	24.50	1.3	1.7	1.3
**6**	F	83	RML	7.48	31.26	0.9	4.0	2.2
**7**	F	73	RML	3.15	19.11	2.5	1.7	1.3
**8**	F	67	RML	6.47	28.87	2.2	4.8	3.4
**9**	F	72	RML	4.42	15.96	2.3	8.7	3.7
**10**	F	76	RML	8.91	37.71	1.4	1.8	1.4
**11**	M	74	RLL	8.54	35.34	1.2	4.6	0.7
**12**	F	68	RLL	7.03	29.35	1.4	8.3	2.2
**13**	F	80	RLL	3.85	19.84	1.5	8.1	5.0
**14**	M	68	RLL	3.85	20.28	1.1	6.4	2.3
**15**	M	82	RLL	5.57	25.52	2.3	9.2	1.9
**16**	F	85	LUL	4.46	18.03	0.9	0.7	1.1
**17**	M	80	LUL	5.58	20.40	2.3	2.8	2.4
**18**	M	80	LUL	7.60	35.73	1.1	1.7	1.3
**19**	M	82	LUL	6.15	29.24	1.1	2.4	1.1
**20**	M	81	LUL	6.15	22.73	1.5	2.7	1.6
**21**	F	76	LLL	5.01	18.26	1.1	1.1	1.3
**22**	F	84	LLL	4.54	22.92	1.8	3.9	2.6
**23**	M	72	LLL	11.43	33.37	3.5	5.1	3.6
**24**	F	82	LLL	14.9	49.72	2.7	5.6	3.1
**25**	M	78	LLL	8.12	35.71	1.8	2.3	2.6

**Table 2:** Motion of the tumor in mm along the 3-axis under the condition of controlled breathing using an abdominal compression device. The results are categorized per individual lobes (5 patients each) as well as all together (25 patients).

	Mean	Std dev	Median	Max	Min
**RUL**					
Lateral Average (mm)	2.2	1.0	2.0	3.8	1.3
Sup/Inf Average (mm)	2.0	0.7	1.8	3.2	1.4
AP/PA Average (mm)	3.1	1.6	3.9	4.6	1.3
**RML**					
Lateral Average (mm)	1.9	0.7	2.2	2.5	0.9
Sup/Inf Average (mm)	4.2	2.8	4.0	8.7	1.7
AP/PA Average (mm)	2.4	1.1	2.2	3.7	1.3
**RLL**					
Lateral Average (mm)	1.5	0.5	1.4	2.3	1.1
Sup/Inf Average (mm)	7.3	1.8	8.1	9.2	4.6
AP/PA Average (mm)	2.4	1.6	2.2	5.0	0.7
**LUL**					
Lateral Average (mm)	1.2	0.2	1.1	1.5	0.9
Sup/Inf Average (mm)	1.8	0.8	1.7	2.7	0.7
AP/PA Average (mm)	1.3	0.2	1.3	1.6	1.1
**LLL**					
Lateral Average (mm)	2.2	1.0	1.8	3.5	1.1
Sup/Inf Average (mm)	3.6	1.9	3.9	5.6	1.1
AP/PA Average (mm)	2.6	0.9	2.6	3.6	1.3
**All Patients**					
**Lateral Average (mm)**	1.8	0.8	1.5	3.8	0.9
**Sup/Inf Average (mm)**	3.8	2.6	2.7	9.2	0.7
**AP/PA Average (mm)**	2.4	1.2	2.2	5.0	0.7
